# TRPM2 promotes pancreatic cancer by PKC/MAPK pathway

**DOI:** 10.1038/s41419-021-03856-9

**Published:** 2021-06-07

**Authors:** Rui Lin, Xunxia Bao, Hui Wang, Sibo Zhu, Zhongyan Liu, Quanning Chen, Kaixing Ai, Baomin Shi

**Affiliations:** 1 General Surgery Department, Tongji Hospital, School of Medicine, Tongji University, Shanghai, 200092 China; 2 School of Life Science, Anhui Medical University, Hefei, 230022 China; 3 School of Life Sciences, Fudan University, Shanghai, 200438 China; 4 Shanghai Pulmonary Hospital, School of Medicine, Tongji University, Shanghai, 200082 China

**Keywords:** Cancer, Molecular biology

## Abstract

The mechanism of pancreatic cancer (PA) is not fully understanded. In our last report, TRPM2 plays a promising role in pancreatic cancer. However, the mechanism of TRPM2 is still unknown in this dismal disease. This study was designed to investigate the role and mechanism of TRPM2 in pancreatic cancer. TRPM2 overexpressed and siRNA plasmid were created and transfected with pancreatic cancer cell line (BxPC-3) to construct the cell model. We employed CCK-8, Transwell, scratch wound, and nude mice tumor-bearing model to investigate the role of TRPM2 in pancreatic cancer. Besides, we collected the clinical data, tumor tissue sample (TT) and para-tumor sample (TP) from the pancreatic cancer patients treated in our hospital. We analyzed the mechanism of TRPM2 in pancreatic cancer by transcriptome analysis, western blot, and PCR. We blocked the downstream PKC/MEK pathway of TRPM2 to investigate the mechanism of TRPM2 in pancreatic cancer by CCK8, scratch wound healing, and transwell assays. Overexpressed TRPM2 could promote pancreatic cancer in proliferation, migration, and invasion ability in no matter the cell model or nude mice tumor-bearing model. TRPM2 level is highly negative correlated to the overall survival and progression-free survival time in PA patients, however, it is significantly increased in PA tissue as the tumor stage increases. The transcriptome analysis, GSEA analysis, western-blot, and PCR results indicate TRPM2 is highly correlated with PKC/MAPK pathways. The experiments of PKC/MEK inhibitors added to TRPM2 overexpressed BxPC-3 cell showed that significant inhibition of PA cells happened in CCK8, transwell, and wound-healing assay. TRPM2 may directly activate PKCα by calcium or indirectly activate PKCε and PKCδ by increased DAG in PA, which promote PA by downstream MAPK/MEK pathway activation.

## Introduction

Pancreatic cancer is one of the most devastating cancer, of which median survival is 3–5 months and the 5-year survival rate is less than 4%^1^. According to the statistics, the incidence and mortality rate gradually increased by 1.3% and 1.25% per year in China in the period of 2004–2015^2^. The real incidence rate must be higher than the statistics because the statistics data^2^ covered only 12% of the nation’s population^3^. Despite the advance in surgical techniques and adjuvant therapy, the prognosis doesn’t have changed much in over four decades. The recent median overall survival have only improved by 5 months on the basis of the new combined therapy for metastasis pancreatic cancer^4^. The molecular mechanism of pancreatic cancer is desperately investigated worldwide nowadays for a better understanding of this dismal disease.

TRPM2 is a kind of ion channel, which contains 1503 amino-acids permeable to Ca^2+^, Na^+^, and K^+^^5^. There were many articles reported the role of TRPM2 in various cancer in recent years. Almasi found that knocking down TRPM2 could downregulate COX4.1, 4.2, and BNIP3 and other autophage related proteins that would cause autophage damage and promote cell death in gastric cancer^6^. Togashi found that downregulating TRPM2 could promote ROS accumulation and DNA damage that could cause inhibition of triple-negative and estrogen receptor-positive breast cancer proliferation by calcium inflow decrease^7^. Klumpp found that the increased level of TRPM2 could promote Bcl (+) T lymph cell in strengthening the ability of DNA repair and cancer cell survival in T cell leukemia patients via P2Y6/P2Y12 pathway^8^. In the last report by our research^9^, TRPM2 played a promising role in promoting pancreatic cancer. Here, we designed new experiments to investigate the role and mechanism of TRPM2 in pancreatic cancer.

## Methods

### Collection of clinical data and specimens

Collected clinical data and pathological specimens of tumor tissue (TT) and paratumor tissue (TP) from 64 patients with pancreatic cancer who were treated in Tongji Hospital Affiliated to Tongji University from May 2017 to May 2019. All the patients enrolled in this research had signed inform consent and didn’t accept any neo-adjuvant treatment before surgery. The patients’ tumor stage were defining according to the pathological results and AJCC TNM staging. All patients were followed up. The end date of follow-up was December 31, 2019. Transcriptome sequencing was performed on the surgical specimens of 12 patients, of which three in each stage of pancreatic cancer. TRPM2 immunofluorescence staining was performed on TT and TP specimens. This study was approved by the Ethics Review Committee of Tongji Hospital Affiliated to Tongji University. All patients signed informed consent.

### Western blot

Part of the tissues of TT and TP in PA patients was homogenized in lysis buffer containing protease and phosphatase inhibitor (Sigma-Aldrich). Protein samples were separated on SDS-PAGE gel and transferred to nitrocellulose membrane. After blocking, incubate with TRPM2, GAPDH, Ras, Raf-1, PSPH, OASL, PKC, METTL3, HIST1H2AE, cPLA, and AQR antibodies (abcam, ab11168, ab9482, ab52939, ab173539, ab211418, ab229136, ab205791, ab195352, ab18255, ab53421, ab205303) at 4 °C overnight. After incubating with the secondary antibody at 20 °C for 1 h, the membrane was washed 3 times. The immune response zone is detected by the ECL detection system. The protein bands were quantified using Image J software (NIH, Bethesda, MD, USA).

### Lentivirus transfection to construct TRPM2 overexpressing/interfering BXPC-3 cells

The pancreatic cancer cell line BXPC-3 cells (CinoAsia Co., Ltd.) were inoculated into a 24-well plate (1 × 10^5^/well), and the added medium ((RPMI 1640; 10% fetal bovine serum, Gibco; Thermo Fisher Scientific, Inc., Waltham, MA, USA)) with a volume of 500 μl. Incubate at 37 °C, 5% CO_2_. The number of cells during lentivirus transfection is about 2 × 10^5^/well. The next day, replace the original medium with 0.5 ml fresh medium containing 10 μg/ml polybrene, and add 10 μl of 1 × 10^8^ TU/ml TRPM2 overexpression/interference lentivirus suspension (Shanghai GenePharma Co., Ltd), After incubating at 37 °C for 24 h, change the medium and replace the virus-containing medium with 1 ml of fresh medium. After 72 h of infection, observe the expression of GFP with a fluorescence microscope, and add medium containing the optimal selection concentration of antibiotics to screen the cells (about a week). The screened cells are further purified using the limiting dilution method, and the cultures are expanded after purification. After the cells have grown to a sufficient number, collect the cells and extract RNA and perform RT-PCR verification. The cells that are verified as positive can be used for subsequent experiments or frozen storage.

### RNA extraction and RT-PCR

Total RNA was extracted from cells and tissues using Trizol reagent, and the quality of extracted RNA was confirmed using NanoDrop 1000. After reverse transcription, a PCR reaction system was prepared, and qPCR analysis was performed by real-time detection system through SYBR green I dye (Takara) detection. According to the PCR standard reaction curve, the original Ct values of the target genes TRPM2, Ras, Raf-1, PSPH, OASL, PKC, METTL3, HIST1H2AE, cPLA, and AQR were obtained, and the 2^−△△Ct^ method was used for semi-quantitative analysis. Table 1 showed the detailed primer sequence of each gene.

**Table 1:** Primer sequence.

Primer	Sequence (5′–3′)
H-TRPM2-F	ATTGTGAAGCGGATGATGAAGGA
H-TRPM2-R	ATGGTGAGGTAGGAGTGGTAGAC
H-GAPDH-F	TGGACCTGACCTGCCGTCTA
H-GAPDH-R	GGAGTGGGTGTCGCTGTTGA
H-Ras-F	CCTTGACGATACAGCTAATTCAGA
H-Ras-R	GCAAATACACAAAGAAAGCCCT
H-Raf1-F	CCTACTGGCTCTGTCCTCTG
H-Raf1-R	CTGATCTCGGTTGTTGATGTGA
H-PSPH-F	GGCTGAAATTCTACTTTAACGGTG
H-PSPH-R	TCCAAATCCAATGAAAGCATCAG
H-OASL-F	AGCAGAGAGTCCCCGATG
H-OASL-R	AGCAGAGAGTCCCCGATG
H-PKC-F	TCATCAACTTAGGCGGTGAGT
H-PKC-R	GTAAGGAGGACAGACAGGAGAG
H-METTL3-F	TGAGTTAGAGAAGAAGTTGCTACA
H-METTL3-R	TCGCTTTACCTCAATCAACTCC
H-HIST1H2AE-F	AAGGCAACTACTCCGAACGA
H-HIST1H2AE-R	TCCGTCTTCTTAGGCAGCAAT
H-cPLA-F	CAGCACCACGCATGACT
H-cPLA-R	ACAGGTAGAGGACAGGCAATG
H-AQR-F	TCATCACCAGTTACCTCCAGTT
H-AQR-R	TTCTGCCTCTCCAAGATTCTGA

### CCK-8 assay

The constructed NC, TRPM2 vector, empty vector, TRPM2 siRNA, and Scramble siRNA BXPC-3 cells were respectively seeded in 96-well culture plates (1 × 10^4^/well) and placed at 37 °C, Incubate overnight in a 5% CO_2_ incubator. Wash the cells inoculated the previous day with PBS twice, then add 100 μl 1640 medium for use. Discard the old solution at 0, 24, and 48 h after changing the medium, add 95 μl of medium and 5ul of CCK-8 to each well of the 96-well plate to be tested, and incubate at 37 °C for 2 h. A microplate reader (BioTek Instruments, Inc., Winooski, VT, USA) was used to measure the OD value of each experimental well at 450 nm, and to detect changes in cell proliferation ability of each group.

### Transwell assay

Inoculate the constructed NC, TRPM2 vector, empty vector, TRPM2 siRNA, and Scramble siRNA group cells into the wells of Matrigel plate containing serum-free RPMI 1640 medium (5 × 10^4^cells/well). The lower well contains 500 µl of complete medium (RPMI 1640 and 10% fetal bovine serum). After incubation at 37 °C for 48 h, gently remove cells that have not migrated through the well with a cotton swab. The cells in the lower chamber were fixed with 5% glutaraldehyde for 10 min and stained with 1% crystal violet in 2% ethanol at room temperature for 20 min, photographed and counted.

### Wound healing assay

The constructed NC, TRPM2 vector, empty vector, TRPM2 siRNA, and Scramble siRNA group cells were inoculated into a 24-well culture plate (1 × 10^5^/well), and placed at 37 °C, 5% CO_2_ for 24 h. Remove the medium and scratch the surface of the inoculated cells with a 10 μl pipette tip and mark it. Wash gently twice with PBS. Add 1 ml RPMI 1640 medium. Photograph the scratches at 0 h and 24 h. The experiment was conducted in three sessions and repeated five times. The distance that the cells migrated to the wounded area during this time was measured. The results are expressed as migration index (the migration distance of cells in the experimental group relative to the migration distance of cells in the NC group).

### Construction of nude mice bearing tumor model

Cells in the logarithmic growth phase were digested with 0.25% trypsin, centrifuged at 800 rpm for 3 min, and the supernatant was discarded to collect the cells. Count the cells after resuspending them in PBS, and adjust the cell concentration to about 6 × 10^7^/ml, put on ice for use.

Prepare 9 male BALB/c nude mice (purchased from Shanghai Experimental Animal Center, Chinese Academy of Sciences) of 4–6 weeks of age with no specific pathogens with an average weight of 17 ± 2 g. Random and equally divided into three groups, three in each group, and inoculated them with BXPC-3 cells in overexpression (OE), normal control (NC), and siRNA interfering (siRNA) groups. We aspirated the cell suspension with a 1 ml syringe (shake the cell suspension before mixing), and slowly injected 150 μl suspension into the subcutaneous area of axilla of nude mice. After the tumor cells were inoculated, the general condition and growth of the nude mice were closely observed. The nude mice were kept in a SPF air clean laminar flow rack at a constant temperature of 22–28 °C and a constant humidity of 45–60%. Measure tumor volume once a week. After 6 weeks, the nude mice were euthanized. The tumors were carefully peeled off and removed. The tumors were placed neatly and photographed. The tumor volume was measured with a ruler and weighed on the scales. Volume formula: volume = long diameter × short diameter^2^ × 1/2. All procedures involved in mice complied with the protocol of Animal Ethics Committee of Tongji Hospital affiliated to Tongji University.

### Immunofluorescence

The TT and TP tissues of PA patients and nude mice were fixed and sectioned. TRPM2 antibody (abcam) was used for immunofluorescence detection to analyze the difference in expression of TRPM2. The slices were scanned for image acquisition and image J was used to analyze the optical density value.

### Transcriptome sequencing analysis

RNA was extracted from TT and TP tissues of PA patients and nude mice, and reverse transcribed into cDNA for PCR amplification. The PCR amplification reaction product was purified and subjected to quality control in a 96-well plate. After dilution, the samples were sequenced using Hiseq2500 sequencer (Illumina, USA). Trimmomatic processed the raw data and used featureCounts software to quantify the expression level of each gene. Perform principal component analysis (PCA) and cluster analysis (HCA) on the samples. And use R package “ComplexHeatmap” and “pheatmap” to draw a heatmap, “stat” package to analyze differentially expressed genes (DEG), with *P* < 0.05 as the standard. RNAseq deconvolution to decipher tumor microenvironment by R package ‘immunedeconv’. Use the ‘Venn Diagram’ online tool to generate DEG intersections, and use the “KEGG profile” for path analysis. DAVIDv6.7 performed gene ontology analysis (GO). Gene Set Enrichment Analysis (GSEA) human mouse pancreatic cancer signal pathway intersection and related genes.

## Results

### Clinical data indicated that high expression levels of TRPM2 in PA patients are associated with poor prognosis

A total of 64 patients with pancreatic cancer were enrolled in this research project, including 38 men and 26 women, whose average age was 63.1 ± 7.4 years. There were 37 patients’ tumors located in pancreatic head, 12 located in uncinate process of pancreas, and 15 located in the body and tail of pancreas. All patients received surgery in our hospital, in which 39 received pancreaticoduodenectomy, 4 received pancreatectomy of middle section, 8 received tail pancreatectomy, and 13 received palliative surgery. All specimens were diagnosed as pancreatic ductal adenocarcinoma by pathologist. According to AJCC TNM stage, the patients were subdivided into 4 different stage groups depending on their pathology results. There were 6 patients with stage I (including IA and IB), 22 patients with stage II (including IIA and IIB), 27 patients with stage III, and 9 patients with stage IV. Table 2 showed the patient baseline characteristics. There were no significant difference for sex (*P* = 0.94), age (*P* = 0.57), and ECOG performance score (*P* = 0.118) among four groups. However, the tumor grade, median survival, and median progression-free survival were statistically significant different among the four groups (*P* < 0.05).

**Table 2:** Patient baseline characteristics (one-way ANOVA, chi-square test, *P < 0.05).

	Stage I	Stage II	Stage III	Stage IV	*P* value*
Sex (%)					0.94
Male	3 (50%)	13 (59%)	16 (59%)	6 (67%)	
Female	3 (50%)	9 (41%)	11 (41%)	3 (33%)	
Age					0.57
Average	66.0	61.7	63.8	62.3*	
Standard deviation	8.0	5.7	9.1	4.8	
ECOG performance score					0.118
0	1	13	9	5	
1	3	8	9	1	
2	2	1	9	3	
3	0	0	0	0	
Tumor grade					<0.001
1	6	16	0	0	
2	0	6	7	0	
3	0	0	20	9	
Median survival	480.5	398	162	58	<0.0001
Median progression-free survival	349	260	50	–	<0.0001

**Fig. 1:**
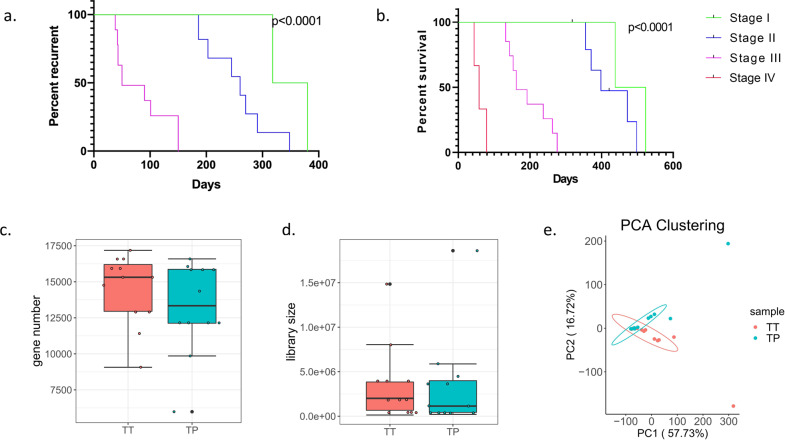
Follow-up data analysis and sequencing data quality control. **a**, **b** The Kaplan–Meier plots showed that the progression-free survival and overall survival of patients decreased significantly as the patients’ tumor stage increased. The significance was tested by Log-rank test. **c** Quality control for patients’ tumor (TT) or para-tumor (PT) tissue: the plot showed no significant different gene number sequenced in two groups. **d** Quality control for patients’ tumor (TT) or para-tumor (PT) tissues: library size sequenced showed no significant difference between two groups. **e** Quality control for patients’ tumor (TT) or para-tumor (PT) tissue: PCA clustering showed that there were significant differences between patients’ TT and TP tissues (12 samples each for TT or TP group).

**Fig. 2:**
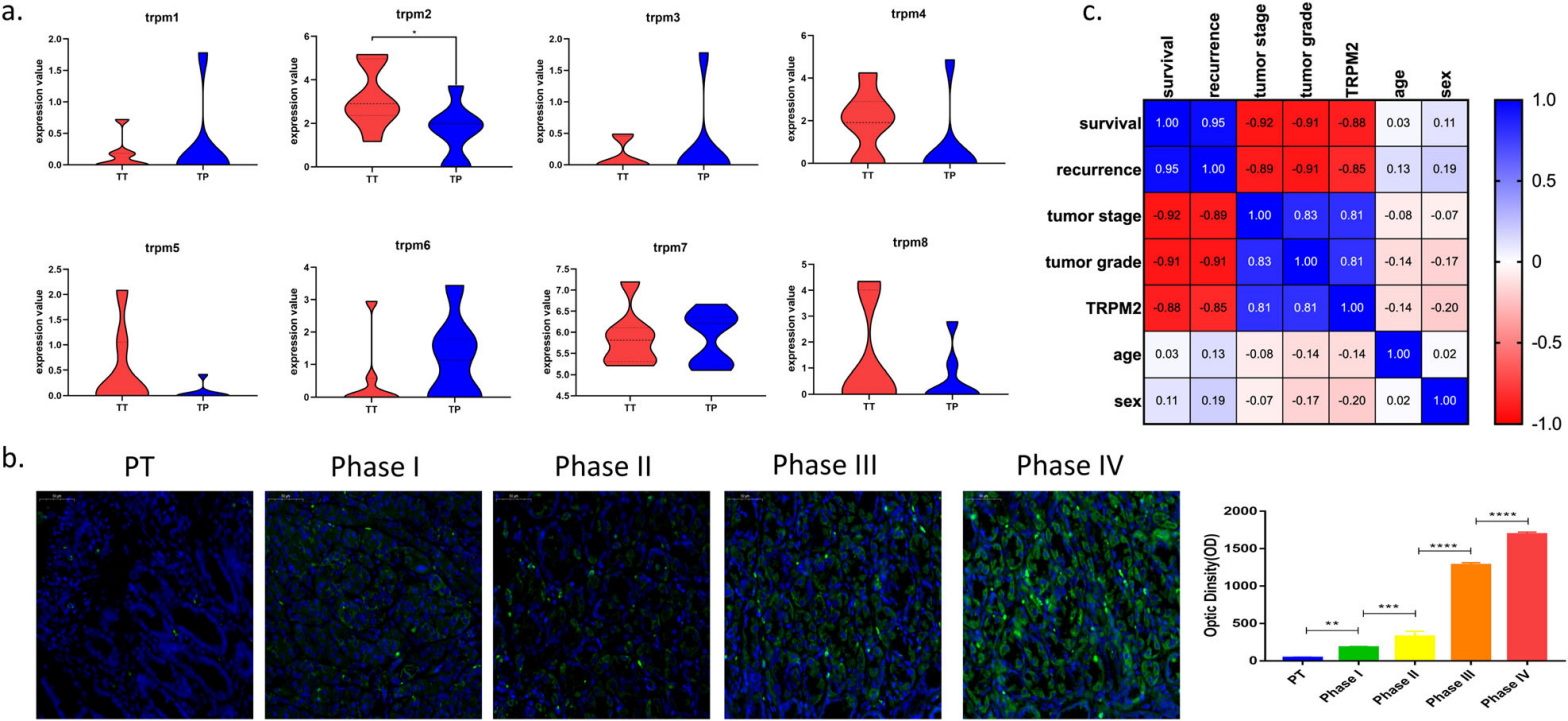
High expression levels of TRPM2 in PA patients are associated with poor prognosis. **a** The mRNA level of TRPM 1–8 in transcriptome sequencing data for TT and TP in PA patients (12 samples each for TT or TP, *P < 0.05). **b** Immunofluorescence staining of TRPM2 in PT and different stages of TT tissues (12 samples for PT and 3 samples each for Stage I to IV TT samples, **P < 0.01, ***P < 0.001, ****P < 0.0001). **c** Pearson’s coefficient in multivariate analysis for patients’ clinical and sequencing data.

The follow-up data showed that the patient’s progression-free survival (PFS, Fig. 1a) and overall survival (OS, Fig. 1b) decreased significantly as the tumor stage increased. After performing RNA sequencing for each tumor and para-tumor specimen, the sequencing data were in good quality control (Fig. 1c–e). We found that TRPM2 was the only molecule of TRPM family that highly expressed in tumor than para-tumor specimens in the RNA level (*P* < 0.05, Fig. 2a). Immunostaining images for different stage groups showed TRPM2 expression in protein level was statistically significantly increasing as the tumor stage increased and tumor specimens had higher level than para-tumor specimens (*P* < 0.05, Fig. 2b). The OD value of TRPM2 expression in immunostaining images were used in the calculation of Pearson coefficient. The Pearson coefficient between TRPM2 level and survival (OS) was −0.88, TRPM2 level and recurrence (PFS) was −0.85 (Fig. 2c). However, the Pearson coefficient between TRPM2 and tumor stage was 0.81 (Fig. 2c).

### TRPM2 promotes the proliferation, migration, and invasion of BxPC-3 pancreatic cancer cells

**Fig. 3:**
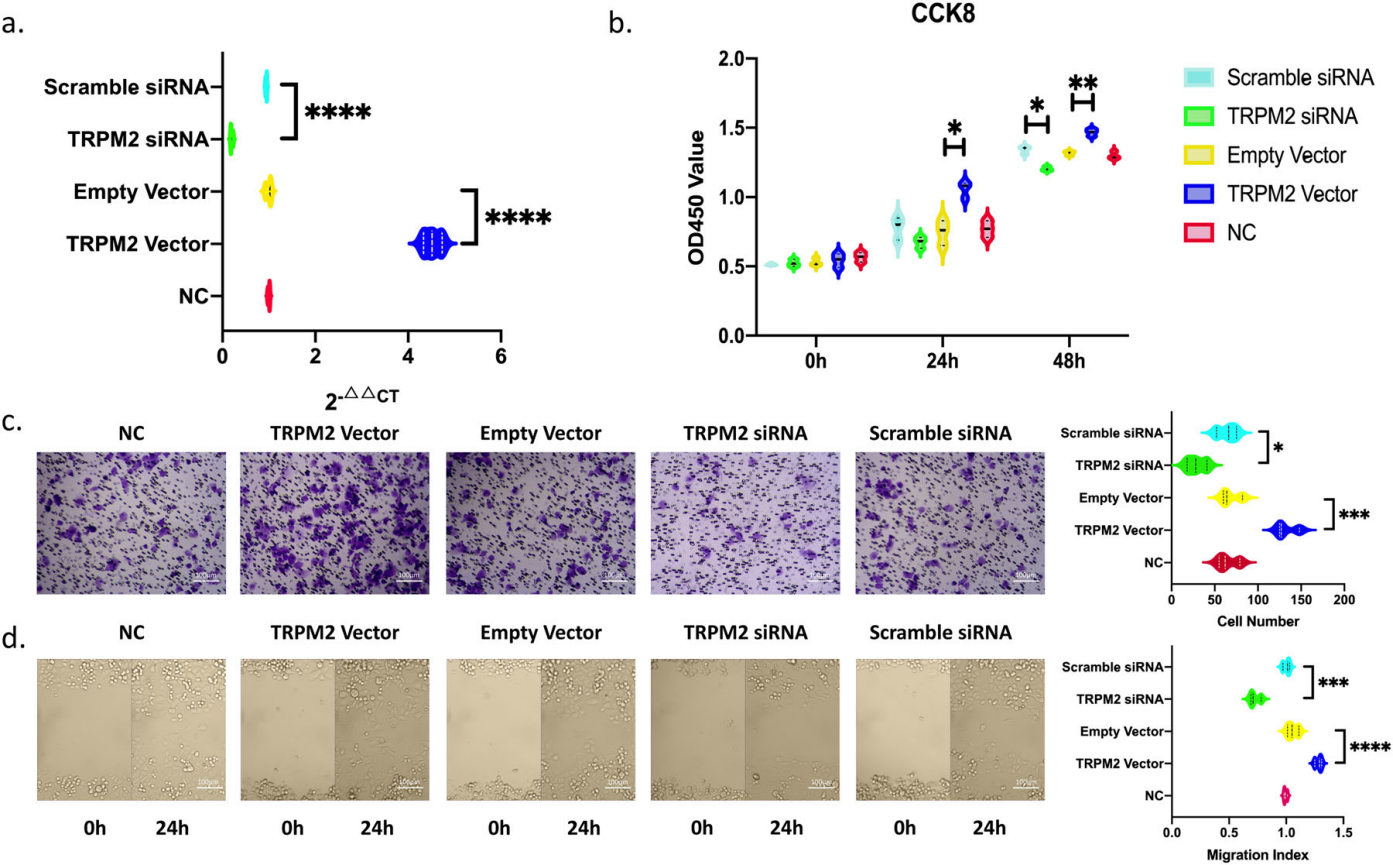
TRPM2 promotes the proliferation, migration and invasion of BxPC-3 pancreatic cancer cells. **a** qRT-PCR results of TRPM2 expression level in different experimental groups of BxPC-3 cells: TRPM2 overexpressed (TRPM2), TRPM2 siRNA, scramble siRNA, empty vector (Empty_Vec), and normal control (NC) groups. ****P < 0.0001. **b** CCK8 assay results of different groups at 0-, 24-, and 48-h time intervals. *P < 0.05, **P < 0.01, siRNA, small interfering RNA; CCK8, cell counting kit-8; OD, optical density. **c** Transwell assay results showed that the TRPM2 overexpression group has a stronger invasive ability and TRPM2 siRNA group has a weaker invasive ability. Images under microscopy (magnification ×100). *P < 0.05. **d** Scratch wound healing assay results. Images of all groups at 0- and 24-h time intervals post injury (magnification ×100). *P < 0.05, **P < 0.01 (3 repeats for each group).

After BxPC-3 cells were transfected with lentivirus, the relative mRNA expression level of TRPM2 in NC, empty vector, Scramble siRNA, TRPM2 siRNA, and TRPM2 vector groups were 1 ± 0.08, 1 ± 0.1, 0.9 ± 0.0.07, 0.3 ± 0.08, 4.43 ± 0.23. The results showed that BXPC-3 cell models with overexpressed and interfered TRPM2 were successfully constructed. The expression level of TRPM2 was significantly higher in TRPM2 overexpressed group than any other group (*P* < 0.0001) and that in TRPM2 siRNA group was significantly lower than any other group (*P* < 0.0001, Fig. 3a).

In CCK-8 assays, 24 h after cell culture for different groups, the OD value of the TRPM2 overexpression group increased significantly compared with other groups (*P* < 0.05). After 48 h, the OD value of the TRPM2 overexpression group increased significantly compared with other groups (*P* < 0.01), and the OD value of the TRPM2 siRNA group decreased significantly (*P* < 0.05). This indicates that TRPM2 has a proliferation-promoting effect on BxPC-3 pancreatic cancer cells (Fig. 3b).

In the Transwell assay, after 48 h of cell inoculation, an average cell count of 5 fields was randomly taken under the ×100 microscope. The cell count of TRPM2 overexpression group was significantly increased compared with other groups (*P* < 0.05), while the cell count of TRPM2 siRNA group was significantly decreased compared with other groups (*P* < 0.05). It showed that TRPM2 increased the invasiveness of BxPC-3 pancreatic cancer cells (Fig. 3c).

In the scratch wound healing assay, the wound healing level was calculated at two time points, 0 and 24 h. As shown in Fig. 3d, the cell migration ability was significantly enhanced in TRPM2 overexpressed group than that of the other groups (*P* < 0.05). However, the cell migration ability in TRPM2 siRNA group was significantly weaker than that of the other groups (*P* < 0.01). It showed that TRPM2 could promote migration of BxPC-3 pancreatic cancer cells.

### TRPM2 function experiment of nude mice tumor-bearing model

**Fig. 4:**
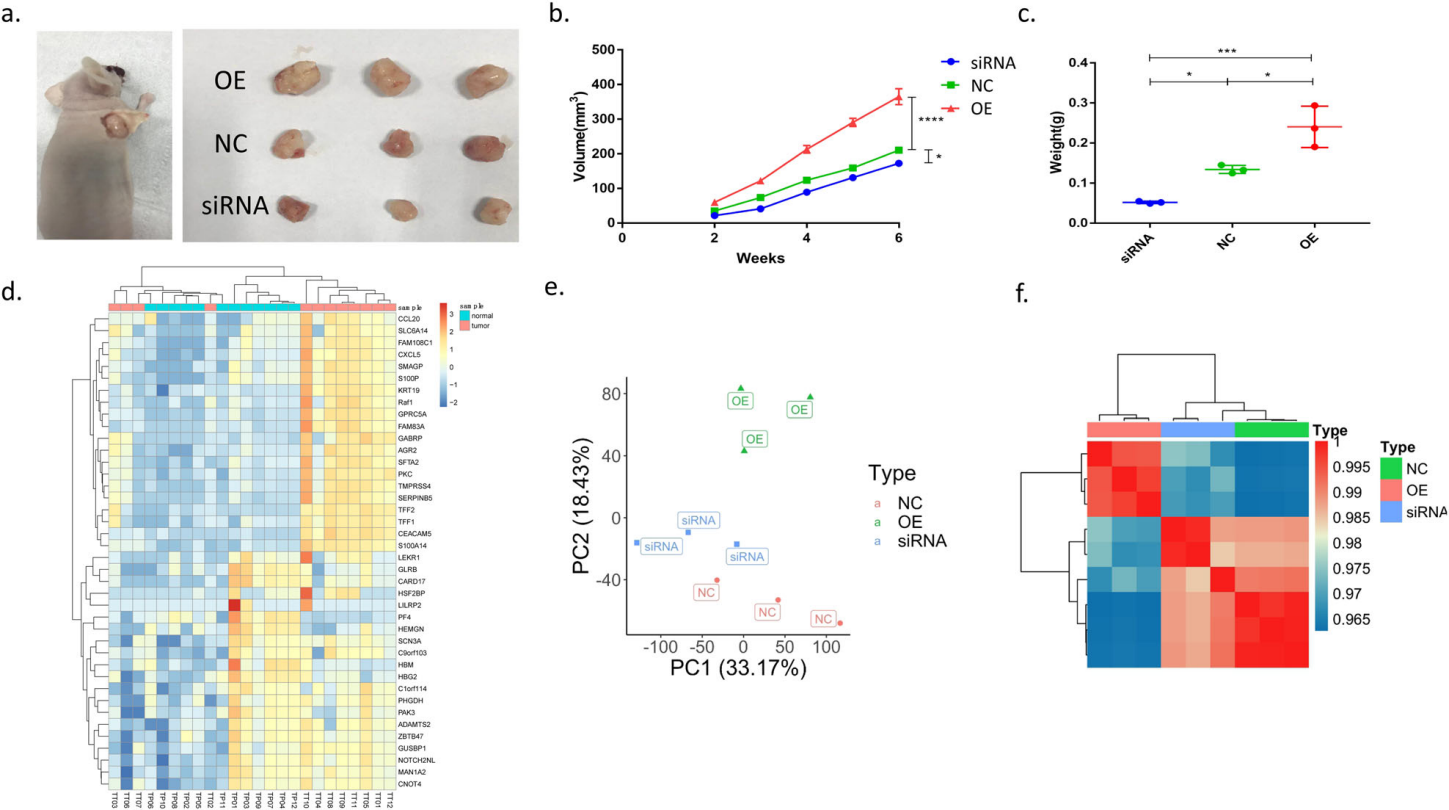
DEGs analysis in samples from tumor-bearing nude mice models and PA patients. **a** Tumors of nude mice (3 repeats in each group). **b** Weekly volume change of nude mice implanted tumors (3 repeats in each group). **c** Weight change of nude mice implanted tumors after six weeks. (OE: TRPM2 overexpression, NC: normal control, TRPM2 siRNA: TRPM2 interference expression group, *P < 0.05, ***P < 0.001, ****P < 0.0001, 3 repeats in each group). **d** Heatmap of top 40 differentially expressed genes (DEG) between TT and TP groups of patients’ PA samples (12 samples each for TT or TP samples). **e** PCA clustering showed significant differences among NC, OE, and siRNA groups (3 samples in each group). **f** Heatmap of DEGs among NC, OE, and siRNA groups (3 samples in each group).

After 6 weeks implantation, the tumors were dissected from the nude mice to measure the volume and weight (Fig. 4a). The volume of the tumors were significantly higher in TRPM2 overexpressed (OE) group than in NC group and TRPM2 siRNA group in each week from the second week to the sixth week after implantation (*P* < 0.0001, Fig. 4b). The weight of the tumors were significantly higher in OE group than in NC (*P* < 0.05) and siRNA group (*P* < 0.001) in the sixth week after implantation (Fig. 4c). It suggested that TRPM2 has the effect of promoting the growth of implanted tumors in nude mice.

### TRPM2 could promote pancreatic cancer through PKC/MEK pathway

We sequenced the transcriptome data in both human tissues (TT vs. TP) and mice implanting tumors (siRNA vs. NC vs. OE). In PCA analysis, we could find that there were significant differences between TT and TP group (Fig. 1e). The top 20 differentially expressed genes (DEGs) between TT and TP groups were displayed in the heatmap (Fig. 4d). Similar outcomes were also seen in tumor-bearing nude mice models (Fig. 4e, f).

**Fig. 5:**
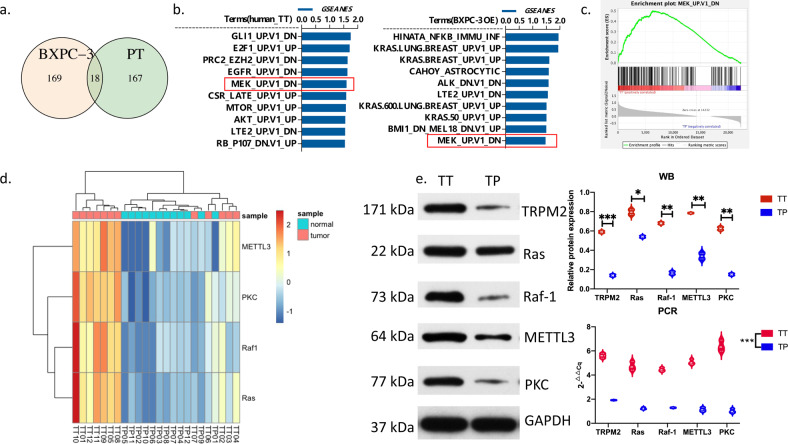
TRPM2 could promote pancreatic cancer through PKC/MEK pathway. **a** Venn graph showed the intersection of signal pathways enriched in tumors of nude mice and human. **b** Top ten standard NES of TT vs. TP in patients and OE vs. NC in nude mice; **c** GSEA analysis of enrichment plot of MEK pathway. **d** Screening of key genes of the MEK pathway signaling pathway in TT vs. TP groups (12 samples each for TT or TP group). **e** Using western blot and qPCR to verify the expression of MAPK/MEK key molecules in patient’s TT and TP groups (*P < 0.05, **P < 0.01, ***P < 0.001, ****P < 0.0001, 12 samples each for TT or TP group).

The DEGs between TT vs. TP groups could enrich in 167 different pathways in the GSEA database (Fig. 5a). Similar outcomes were found in DEGs between OE vs. siRNA groups in nude mice, in which 169 different pathways were enriched in the GSEA database (Fig. 5a). There were 18 common pathways between human and mice samples (Fig. 5a).

The top 10 pathways from 18 common pathways between human and mice samples were listed in Fig. 5b. As shown in the figure, MEK pathways ranked the top 10 in both human and mice samples (Fig. 5b, c).

The downstream pathways of TRPM2 from KEGG database was shown in Supplementary Fig. 1. The TRPM2 may promote pancreatic cancer through PKC/MEK pathways.

**Fig. 6:**
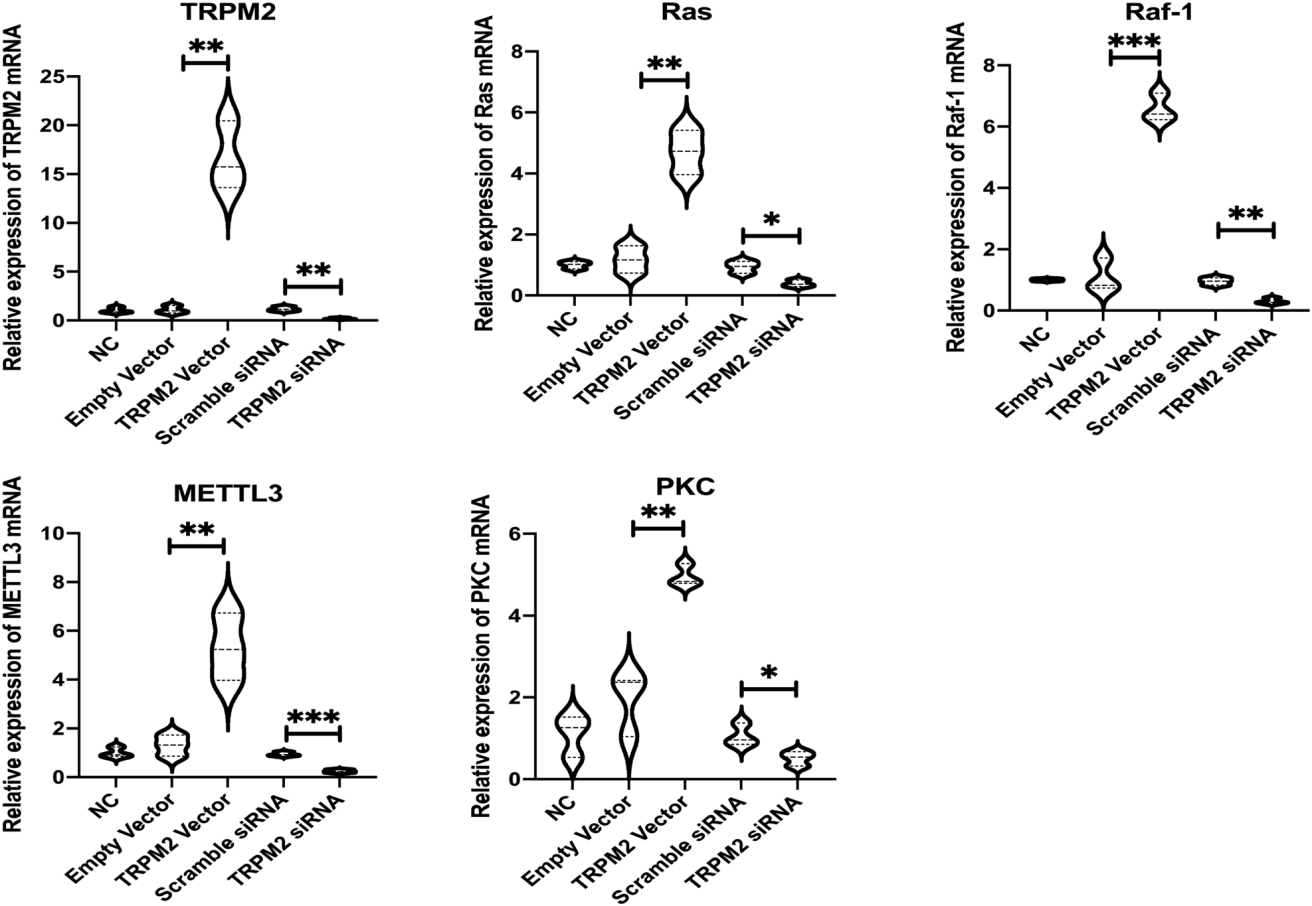
Expression of MAPK/MEK pathway key molecules in BxPC-3 stable transfection groups. NC, normal control; Empty Vector, BxPC-3 without any plasmid transfection; TRPM2 vector, TRPM2 overexpression group; scramble siRNA, BxPC-3 cells with scramble sequence of small interfering RNA transfection group; TRPM2 siRNA, BxPC-3 cells with TRPM2 small interfering RNA transfection group. **P < 0.01, ***P < 0.001, three repeats in each group.

For identifying the mechanism of TRPM2/PKC/MEK, we picked out four DEGs, including Ras, Raf-1, METTL3, and PKC, in the PKC/MEK pathways from human sequencing data and drew the heatmap (Fig. 5d). In the heatmap, these 4 genes were expressed significantly higher in the TT than TP tissues. There were significant differences between TT and TP tissues in each of the four genes no matter in protein (western blot) or mRNA (qPCR) level (Fig. 5e). Similar results were also found in BxPC-3 models. Each of the 4 genes plus TRPM2 were significantly higher expressing in TRPM2 overexpressed group than NC, empty vector, and scramble siRNA groups in BxPC-3 cell models (Fig. 6). Each of the 4 genes plus TRPM2 were significantly lower expressing in TRPM2 siRNA group than NC, empty vector, and scramble siRNA groups in BxPC-3 cell models (Fig. 6).

**Fig. 7:**
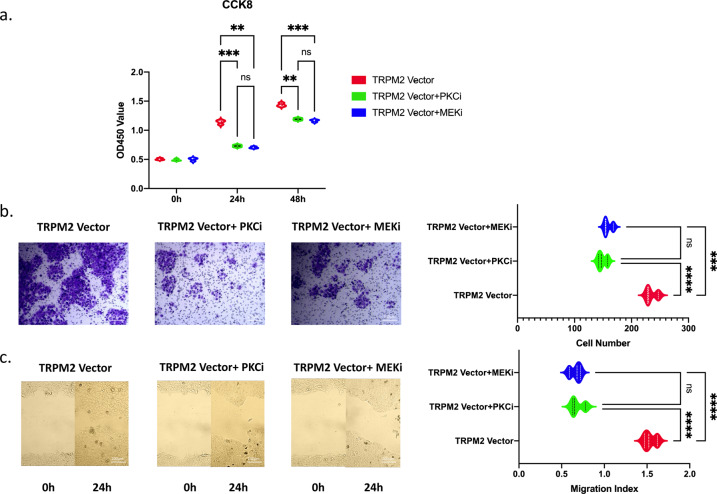
Using PKC inhibitor sotrastraurin (PKCi) and raf-1 inhibitor sorafenib (MEKi) to test the proliferation, invasion, and migration abilities of TRPM2 overexpressed (TRPM2 Vector) BxPC-3 cells. **a** CCK8 results. **b** Transwell results. **c** Scratch wound-healing assay results. **P < 0.01, ***P < 0.001 (three repeats for each group).

Then we used pan PKC inhibitor Sotrastaurin^10–12^ and Raf-1 inhibitor sorafenib^13^ to test the ability of proliferation, invasion, and migration in TRPM2 overexpressed BxPC-3 cells by CCK8, Transwell, and wound-healing assay. As shown in Fig. 7a, the proliferation ability of PA cells were significantly inhibited by Sotrastaurin or sorafenib. So as the transwell (Fig. 7b) and wound-healing (Fig. 7c) assays, the invasion and migration ability were also significantly inhibited by Sotrastaurin and sorafenib.

The above results indicate that TRPM2 was closely related to its downstream regulatory pathway PKC/MEK in promoting the proliferation and invasiveness of human pancreatic cancer cells.

## Discussion

Transient receptor potential (TRP) channels are a cluster of ion channels that have many physiological functions^14^. The subfamily of TRPM (Melastatin) participate in modulation of cell proliferation and survival^15^. One of these is TRPM2, which is the second member of the TRPM subfamily to be identified. Human TRPM2 is permeable to Ca^2+^, Na^+^, and K^+^ which consists of 1503-amino acid^5^.

TRPM2 is highly expressed in many kinds of cancer, like gastric cancer^6^, breast cancer^16^, prostate cancer^17^, and lung cancer^18^. It was reported that TRPM2 could promote cancer proliferation through the regulation of oxidative stress^19^, autophagy^6^, and mitochondrial function^20^.

In 2018, our group first report the role of TRPM2 in pancreatic cancer^9^. Plus the results in this article, we have demonstrated that TRPM2 could promote proliferation and invasion ability in vitro in both PANC-1 and BxPC-3 (Fig. 3) pancreatic cancer cell lines. The nude mice tumor-bearing model results in this article is another evidence that support TRPM2’s promotion effect in pancreatic cancer (Fig. 4a–c).

We found that TRPM2 was significantly higher expressed in human pancreatic cancer tissue than in para-tumor tissue in both mRNA level (Fig. 2a) and protein level (Fig. 2b). The level of TRPM2 also increased as the patients’ TNM stage increased (Fig. 2b), meanwhile, the patients’ overall survival (OS) period significantly shortened as the TNM stage (Pearson’s coefficient = −0.92) and TRPM2 level (Pearson’s coefficient = −0.88) increased in pancreatic cancer patients (Fig. 2c). The progression-free survival (PFS) period also significantly shortened as the TNM stage (Pearson’s coefficient = −0.89) and TRPM2 level (Pearson’s coefficient = −0.85) increased in pancreatic cancer patients (Fig. 2c).

The gene set enrichment analysis (GSEA) of human and nude mice PA tissue found that TRPM2 was highly correlated to MEK pathway (Figs. 4d–f, 5a–c). According to KEGG database, PKC/MEK was downstream pathway of TRPM2 (Supplementary Fig. 1). The mRNA seq (Fig. 5d), western blot, and qPCR (Fig. 5e) of 4 different genes in the PKC/MEK pathway found that these genes plus TRPM2 were significantly highly expressed in TT than in TP in PA patients. The BxPC-3 cell model also demonstrated TRPM2 overexpressing was closely related to PKC/MEK pathway in pancreatic cancer (Fig. 6). We knocked down the downstream PKC and Raf-1 in TRPM2 overexpressed BxPC-3 cells. The results (Fig. 7a–c) showed that the proliferation, invasion, and migration abilities were significantly inhibited in PA cells. That was why we concluded TRPM2 could promote PA through PKC/MEK pathway.

MAPK/MEK, which contained molecules like Raf, MEK1/2, and ERK1/2^21^, was the downstream pathway of KRAS in PA. KRAS could activate Raf directly, which subsequently promote the development of PA by phosphorylating MEK or ERK in mice^22^. Some researchers tried to block MEK’s activation to inhibit PA development. But AKT/PI3K pathway was accidentally activated after MEK inhibition and resulted in promoting PA^23^. If inhibited MEK and PI3K simultaneously, the apoptosis of PA could be increased in vitro assays^24^. But this combined inhibition could only work in PA when KRAS was mutated^25^. Nowadays, we could find abundant publications about KRAS/MEK’s role in PA. But we still can’t translate them into clinical practice. The exact mechanism still needs to be clarified further.

Protein kinase C (PKC), which also promotes PA development, consists of classical PKC (cPKC), novel PKC (nPKC), and atypical PKC (aPKC)^26^. The subfamily was divided by C1 and C2 domain in the molecular structure^27^. cPKC contains both C1 and C2 domain, which could be activated by diacylglycerol (DAG) and Ca^2+^. nPKC contains only C1 domain. It could be activated only by combining DAG. aPKC contains neither C1 nor C2 domain. It could not be activated by DAG or Ca^2+^^27^.

PKCα, PKCβ, and PKCγ belongs to cPKC subfamily, in which PKCα could promote proliferation and metastasis ability of PA by activating Raf-1^28^. PKCε, PKCδ, PKCη, and PKCθ belongs to nPKC subfamily, in which PKCε and PKCδ could also promote PA by activating Raf-1^29–31^. PKCζ and PKCι belongs to aPKC subfamily, in which PKCι could promote PA by activating Rac1-MAPK pathway^32^.

According to the literature review above, TRPM2 is an ion channel on the cell membrane permeable to Na +, K +, and Ca2 +. Because TRPM2 is highly expressed in PA, calcium level is also elevated. We postulated that calcium could activate PKCα to promote PA further. On the other hand, DAG is an important second messenger in cells. It could be produced in the process of degradation of phospholipids in cell membrane by phospholipase C (PLC)^33^. So we postulated that elevated calcium level in PA could also activate PLC to increase DAG level. Then increased DAG could activate PKCε and PKCδ to promote PA further. PKCι couldn’t be activated by calcium and DAG. So it is not TRPM2 dependent.

Here, we could draw a cautious postulation that TRPM2 could directly activate PKCα by calcium or indirectly activate PKCε and PKCδ by increased DAG in PA, which promote PA by downstream MAPK/MEK pathway activation. The exact mechanism of how TRPM2 promoted PA development in patients still need further investigation. Our study still needs to be improved due to funding constraints. For example, we did not focus on detailed differences between cell models and human samples in our selection of cell types (e.g., BxPC3 cell line lacks KRas mutation). A single cell line may result in less comprehensive conclusions. We will use more cell lines and collect more patients’ samples to verify our postulation in the near future.

## Conclusion

**Fig. 8:**
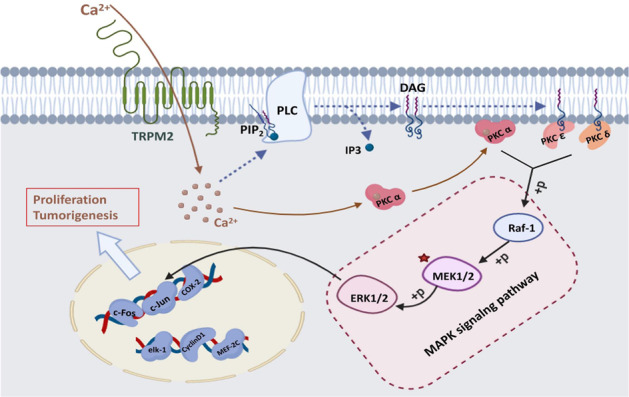
The graph indicates the mechanisms of how TRPM2 promote pancreatic cancer proliferation and tumorigenesis. The increased level of calcium in pancreatic cancer cell could directly activate PKCα, which in turn cause MAPK pathway activation. On the other hand, the increased calcium level could activate MAPK pathway by indirectly activate PKCε and PKCδ.

TRPM2 may directly activate PKCα by calcium or indirectly activate PKCε and PKCδ by increased DAG in PA, which promote PA by downstream MAPK/MEK pathway activation (Fig. 8).

## Supplementary information

Supplementary figure

Supplementary figure legend
