# Supplementary figures and images for: TRPM2 promotes pancreatic cancer by PKC/MAPK pathway
Source: Cell Death Dis. 2021 Jun 7;12(6):585. doi: 10.1038/s41419-021-03856-9 (PMC8184946; doi:10.1038/s41419-021-03856-9)

# OXYTOCIN SIGNALING PATHWAY

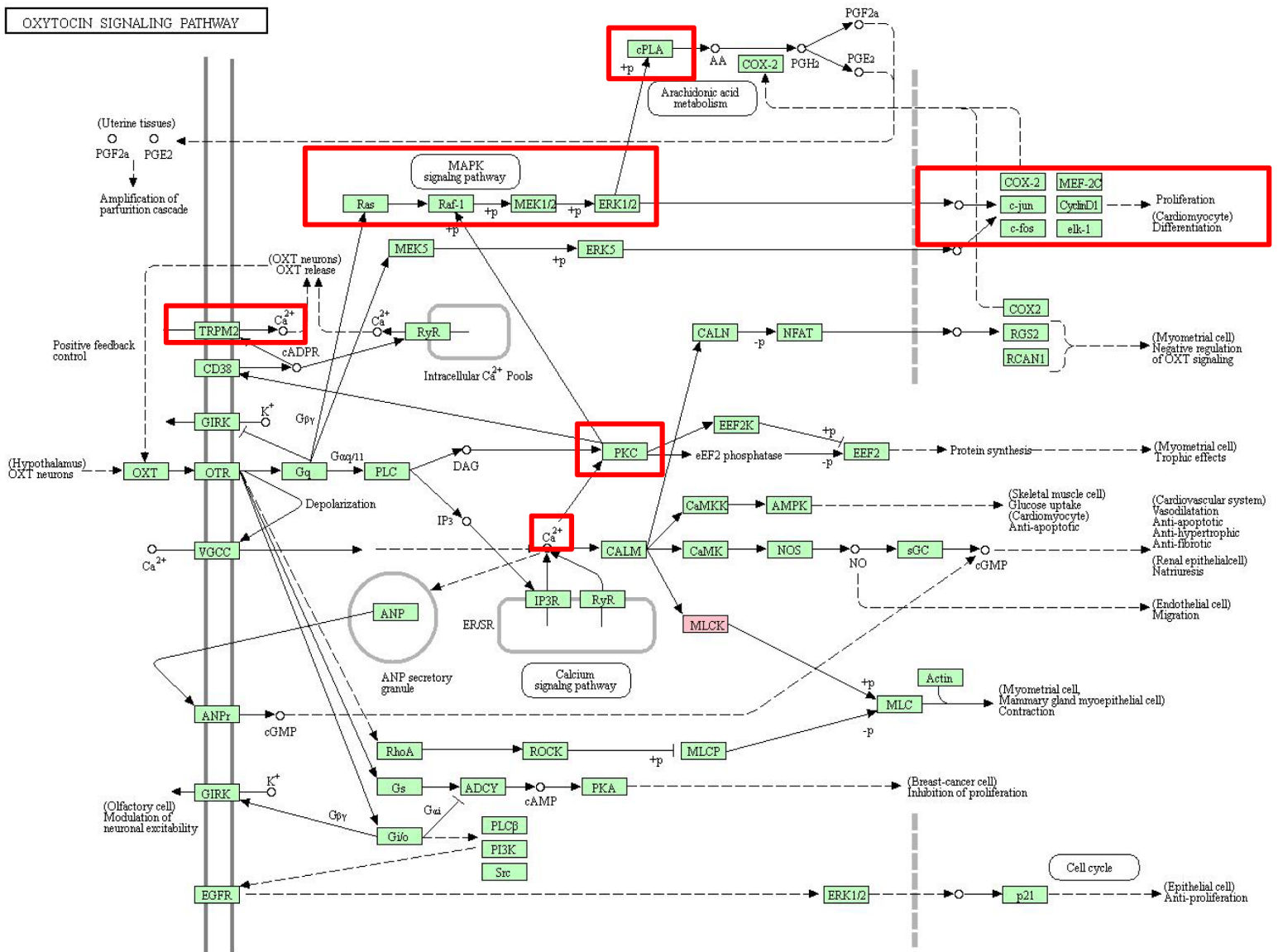

Supplement: Supplementary file 1 — Supplementary figure [file 41419_2021_3856_MOESM1_ESM.pdf]
