# Supplementary material for: TRPM2 promotes pancreatic cancer by PKC/MAPK pathway
Source: Cell Death Dis. 2021 Jun 7;12(6):585. doi: 10.1038/s41419-021-03856-9 (PMC8184946; doi:10.1038/s41419-021-03856-9)
Supplement: Supplementary file 2 — Supplementary figure legend [file 41419_2021_3856_MOESM2_ESM.docx]

Supplementary fig. 1. Downstream pathways of TRPM2 in KEGG database. The red-square marking pathway coincides with the pathway we found in this article. TRPM2 could increase calcium inflow to activate PKC. MAPK pathway is the downstream pathway of PKC which could promote cell proliferation.
